# The Long Road Toward COVID-19 Herd Immunity: Vaccine Platform Technologies and Mass Immunization Strategies

**DOI:** 10.3389/fimmu.2020.01817

**Published:** 2020-07-21

**Authors:** Lea Skak Filtenborg Frederiksen, Yibang Zhang, Camilla Foged, Aneesh Thakur

**Affiliations:** ^1^Department of Pharmacy, Faculty of Health and Medical Sciences, University of Copenhagen, Copenhagen, Denmark; ^2^Department of Pharmaceutics, School of Pharmacy, Jiangsu University, Zhenjiang, China

**Keywords:** coronavirus, SARS-CoV-2, COVID-19, vaccine, immunopathology, immune response, animal models, herd immunity

## Abstract

There is an urgent need for effective countermeasures against the current emergence and accelerating expansion of coronavirus disease 2019 (COVID-19), caused by severe acute respiratory syndrome coronavirus 2 (SARS-CoV-2). Induction of herd immunity by mass vaccination has been a very successful strategy for preventing the spread of many infectious diseases, hence protecting the most vulnerable population groups unable to develop immunity, for example individuals with immunodeficiencies or a weakened immune system due to underlying medical or debilitating conditions. Therefore, vaccination represents one of the most promising counter-pandemic measures to COVID-19. However, to date, no licensed vaccine exists, neither for SARS-CoV-2 nor for the closely related SARS-CoV or Middle East respiratory syndrome-CoV. In addition, a few vaccine candidates have only recently entered human clinical trials, which hampers the progress in tackling COVID-19 infection. Here, we discuss potential prophylactic interventions for SARS-CoV-2 with a focus on the challenges existing for vaccine development, and we review pre-clinical progress and ongoing human clinical trials of COVID-19 vaccine candidates. Although COVID-19 vaccine development is currently accelerated via so-called fast-track programs, vaccines may not be timely available to have an impact on the first wave of the ongoing COVID-19 pandemic. Nevertheless, COVID-19 vaccines will be essential in the future for reducing morbidity and mortality and inducing herd immunity, if SARS-CoV-2 becomes established in the population like for example influenza virus.

## Introduction

Coronavirus disease 2019 (COVID-19), caused by the novel severe acute respiratory syndrome coronavirus 2 (SARS-CoV-2), emerged in December 2019 in Wuhan, China, and rapidly spread globally due to high transmissibility and pathogenicity (1, 2). According to the World Health Organization (WHO), the disease has infected more than 9.0 million people across 216 countries and territories as of June 23rd 2020, with evidence of ongoing local transmission (3). In most cases, the symptoms of COVID-19 are mild and include fever, cough, and shortness of breath. However, in certain cases, the disease develops into severe pneumonia and multiple organ failure, primarily in elderly and patients with other underlying diseases or conditions, and it has a mortality rate of ~3.7% (4). On January 30th 2020, WHO declared COVID-19, a public health emergency of international concern. At present, the understanding of the pathogenesis of and immunity against COVID-19 is incomplete, and there is no approved therapy or prophylaxis against the disease. Hence, there is an urgent need to develop both new therapeutics and prophylactics to contain SARS-CoV-2, given the pandemic spread and the associated enormous global humanitarian and economic losses.

Vaccines represent one of the most successful and cost-effective health interventions in human history (5). According to the WHO, global vaccination programs save up to 2–3 million lives each year by priming the immune system to protect the host against potential pathogens, who would otherwise significantly challenge global health and economy (6). Besides providing individual protection, vaccination programs also aim for so-called *population* or *herd immunity*, i.e., immunization of a large proportion of the population to protect the non-vaccinated, immunologically naïve, and immunocompromised individuals by reducing the percentage of vulnerable hosts to a level below the transmission threshold (7). For example, a global immunization coverage of more than 80% against smallpox virus has reduced the transmission rates to uninfected individuals to such low levels that the virus has been eradicated (6). For measles, 91–94% of a population must be vaccinated to achieve herd immunity and prevent new measles outbreaks (8). Likewise, a threshold of 80–85% is now the target for global eradication of poliovirus (6). These examples illustrate well that the threshold for vaccination-induced herd immunity is pathogen specific. A threshold value of ~67% is estimated to be sufficient for achieving herd immunity against SARS-CoV-2, assuming that the basic reproductive number (*R*_0_) of the virus is three, i.e., one infected individual infects three new individuals (9). Based on this estimate, ~5.3 billion vaccine doses are required for a single-dose vaccine, or possibly 12–16 billion in case of a multi-dose vaccine. Therefore, it is clear that inducing herd immunity by mass vaccination would be an incredibly powerful tool to contain the COVID-19 pandemic, but it also represent a massive challenge.

The urgent need for safe and efficacious vaccines against COVID-19 has accelerated the development of a number of vaccine candidates, of which a few have already progressed into phase I/II clinical testing. Globally, academic partners are collaborating with vaccine manufacturers to exploit a number of different novel and established vaccine development and manufacturing platforms in the design of COVID-19 vaccines at an unprecedented pace. Here, we review these global efforts with focus on the vaccine candidates in preclinical and clinical development. We also describe the characteristics of the SARS-CoV-2 virus and the immunopathology of the infection, and discuss the host immune response and animal models.

## Characteristics of SARS-COV-2

### Genome and Virion

**Figure 1:**
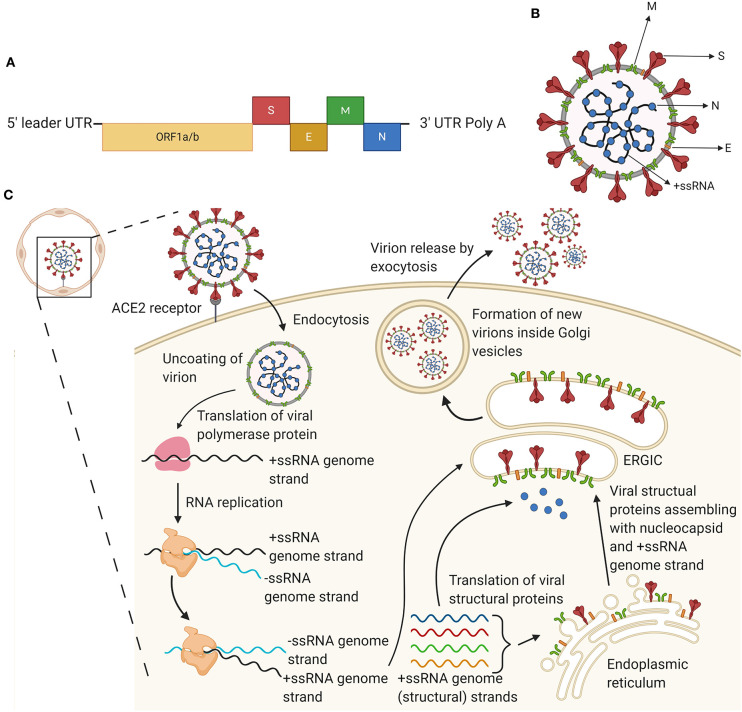
The genome, virion, and replication of severe acute respiratory syndrome coronavirus 2 (SARS-CoV-2). **(A)** Schematic diagram of the SARS-CoV-2 genome. Approximately two-thirds of the positive single stranded RNA genome encodes a large polyprotein (ORF1a/b; nude). The last third of the genome proximal to the 3′-end encodes four structural proteins, i.e., the spike (S), envelope (E), membrane (M), and nucleocapsid (N) proteins (red, orange, green, and blue, respectively). The colors of the structural proteins are consistent in this figure. **(B)** Schematic diagram of the SARS-CoV-2 virion. The virion displays a nucleocapsid composed of genomic RNA (+ssRNA) and N protein, which is enclosed inside the virus envelope consisting of S, E, and M proteins. **(C)** Schematic overview of the life cycle of SARS-CoV-2 in host cells. The life cycle is initiated upon binding of the S protein to angiotensin-converting enzyme 2 (ACE2) on host cells, e.g., epithelial cells in the alveoli. After receptor binding, a conformational change in the S protein facilitates viral endocytosis and envelope fusion with the cell membrane. Subsequently, viral genomic RNA is released into the host cell, and viral +ssRNA is translated into viral polymerase encoded by the genome, which initiates replication of +ssRNA to –ssRNA and further produces a series of genomic and subgenomic mRNAs. These are translated into viral proteins, which are subsequently assembled with genomic RNA into virions in the endoplasmic reticulum (ER) and the ER-Golgi intermediate compartment (ERGIC) to form mature virions that are trafficked via Golgi vesicles out of the cell by exocytosis. Created with Biorender.com.

Coronaviruses (CoVs) constitute a genus in the *Coronaviridae* family, which are pleomorphic enveloped viruses (10). The *Coronaviridae* are classified into four subgroups, including (i) alpha (α), (ii) beta (β), (iii) gamma (γ), and (iv) delta (δ) coronaviruses. The former two subtypes usually infect mammals, whereas the latter two subtypes predominantly infect birds. The novel SARS-CoV-2 is a member of the β subgroup, along with SARS-CoV and Middle East respiratory syndrome (MERS)-CoV (11, 12). All CoVs are enveloped, positive single-stranded RNA viruses, and they have relatively large RNA genomes ranging from 26 to 32 kilobases (kb) (12). The genome of SARS-CoV-2 contains a 5′ cap structure and a 3′ poly(A) tail, allowing it to serve as messenger RNA (mRNA) for translation of the replicase polyproteins (Figure 1A). The open reading frames (ORFs) 1a/b occupy two-thirds of the genome (~20 kb) and encode the replicase polyproteins. The replicase polyproteins include the 1–16 non-structural proteins (nsps1-16), which are responsible for (i) viral replication, (ii) RNA-dependent RNA-polymerase activity, (iii) helicase activity, and (iv) assembly of virus replication structures (11). The majority of the remaining one-third of the genome encodes structural and accessory proteins (11–13). Coronaviruses contain four major structural proteins, i.e., the spike (S), envelope (E), membrane (M), and nucleocapsid (N) proteins (Figure 1B). The 5′ end of the genome contains a leader sequence and an untranslated region (UTR), including structures required for RNA replication and transcription. The 3′ UTR also encodes RNA structures required for replication and synthesis of viral RNA. The genomic sequence of CoV is 5′-leader-UTR-replicase-S-E-M-N-3′-UTR-poly(A) tail with accessory genes interspersed between the structural proteins at the 3' end of the genome (13). Interestingly, the accessory genes encoding the ORF3b, ORF6, and N proteins are interferon (IFN) antagonists, which act on the type I IFN pathway, either by inhibiting transcription or by acting on effector mechanisms, and they modulate the host innate immune response (14, 15). Like other coronaviruses, SARS-CoV-2 virions are spherical in shape with a diameter of 65–125 nm (16), and the most prominent features include the spikes projections emanating from the surface of the virions. These spike projections give the virus the resemblance of a crown, hence the name coronavirus (12, 17). The S protein represents the *key* on the virion, which binds by *locking* into its receptor on a host cell. The N proteins hold the RNA genome, and together, the S, E, and M proteins constitute the viral envelope (18).

It is crucial to investigate the impact of mutations in the major antigenic proteins of SARS-CoV-2 when developing vaccines and vaccination strategies against SARS-CoV-2. The S protein is the most commonly used SARS-CoV-2 virus protein for vaccine development (19). Recently, 149 mutation sites have been identified across the genome from 103 sequenced strains of SARS-CoV-2 (20), indicating that there is a high mutation rate within these strains. SARS-CoV-2 strains in this study had evolved into two different subtypes (L, which is a more aggressive type and S, which represents a less aggressive type) with great differences in geographical distribution, transmission ability, and severity of disease (20). Hence, these differences also complicate vaccine design (20). In another study, of the 144 sequences of global SARS-CoV-2 strains, two subtypes SARS-CoV-2a (China strains) and SARS-CoV-2b (USA strains) were identified, which differ only by a novel synonymous mutation of position D614G in the S protein and display different antigenicity (21). Domains containing this mutation point have been confirmed to represent B-cell epitopes (21). Further, it has been reported that the antigenic indexes were reduced more for SARS-CoV-2b than for SARS-CoV-2a (21). These results indicate that different subtypes may display different antigenicity and that vaccine development may benefit from a strategy focused on targeting multiple subunits of the virus (21).

### Viral Replication

SARS-CoV receptor recognition and attachment is initiated via interactions between the S protein and the human angiotensin-converting enzyme 2 (ACE2) expressed by cells in (i) vascular endothelia, (ii) renal and cardiovascular tissue, (iii) epithelia of the airways, small intestine, and testes, and (iv) lung parenchyma [(11, 13); Figure 1C]. The S protein of SARS-CoV-2 has been shown to engage with a comparable affinity with human ACE2 as the SARS-CoV S protein (16). Due to the genomic resemblance between the novel SARS-CoV-2 and SARS-CoV, SARS-CoV-2 is expected to display a pathogenesis, which is similar to that of SARS-CoV. ACE2 is suggested to play a protective role in inflamed lung tissue, and the binding of the SARS-CoV S protein to ACE2 is assumed to contribute to disease severity (11). Following receptor binding and attachment, SARS-CoV-2 gains access to the host cell cytosol. This is accomplished by cleavage of the S protein by cathepsin, transmembrane protease serine 2 (TMPRRS2) or another protease, followed by fusion of the viral and cellular membranes (12, 13). The S protein of SARS-CoV-2 has been shown to contain a furin cleavage site between the two polypeptides referred to as the S_1_ and S_2_ subunits, which is not present in the S protein of SARS-CoV (16). An additional cleavage of the S_2_' subunits is important for separating the receptor-binding domain (RBD) and the fusion domains, and for exposing the fusion peptide (13, 16). Subsequently, the fusion peptide is inserted into the membrane, followed by the formation of an antiparallel six-helix bundle, which allows mixing of cellular and viral membranes, eventually resulting in fusion and release of the viral genome into the cytosol (13). The next step for SARS-CoV replication is translation of the replicase gene from the virion genomic RNA. The replicase gene encodes two large ORFs, i.e., *rep1a* and *rep1b*, which code for the co-terminal polyproteins pp1a and pp1b, respectively (13). These polyproteins are subsequently cleaved into nsps1-16, which assemble into the replicase-transcriptase complex, where RNA synthesis takes place. Ultimately, nsps1-16 facilitate RNA replication and transcription of the sub-genomic RNAs (11, 13). Viral RNA synthesis follows the translation and assembly of viral replicase complexes. Both genomic and subgenomic RNAs are produced by viral RNA synthesis through negative-strand intermediates (12, 13). Subgenomic RNAs serve as mRNAs for the structural and accessory genes. After replication and subgenomic RNA synthesis, the structural proteins S, E, and M are translated and inserted into the endoplasmic reticulum (ER) (12). Here, they are transported into the ER-Golgi intermediate compartment (ERGIC), where viral genomes become encapsulated by the N protein, resulting in the formation of mature virions (13). The virions are subsequently transported in vesicles to the cell surface and released through exocytosis, thereby contributing to the generation of new virions able to infect host cells and promote human-to-human transmission (11, 12).

## COVID-19 Disease

### Transmission

According to the WHO, SARS-CoV-2 has killed more than 469,159 and infected over 8,974,795 individuals globally by June 23rd 2020. Hence, SARS-CoV-2 has a higher transmission rate compared to SARS-CoV in 2002–2003, which infected 8,098 and killed more than 700 individuals. This may be the result of genetic recombination in the RBD of the S protein, thus enhancing the transmission ability of SARS-CoV-2 (12). For preventive strategies against SARS-CoV-2, it is important to determine the source of origin and transmission of the virus. The outbreak arose at the Huanan Seafood market in the city of Wuhan, China, and SARS-CoV-2 rapidly infected more than 50 individuals. At this market, which is now closed, live animals were frequently sold, e.g., bats, birds, frogs, rabbits, and snakes. Genomic analyses revealed similarities between SARS-CoV-2 and SARS-like bat viruses, hence bats are suspected to be reservoirs for SARS-CoV-2 (1). In another study, the origin of SARS-CoV-2 has been associated with Pangolin-CoV, because Pangolin-CoV was found to be 91.02 and 90.55% identical to SARS-CoV-2 and Bat-CoV, respectively (22). Close contact with these infected animal reservoirs is the major cause of animal-to-human SARS-CoV-2 transmission (23), which eventually leads to a rapid human-to-human transmission (12, 24). Respiratory droplets and contact transmission are considered as the main transmission routes for human-to-human transmission, and aerosol spread is suspected to be another important transmission route (18). The stability of SARS-CoV-2 on various surfaces has been investigated, indicating that aerosol and fomite transmission of SARS-CoV-2 is plausible, because the virus remains viable and infectious in aerosols for several hours and even up to days on surfaces (25). Pharyngeal virus shedding and active virus replication in the upper respiratory tract has been confirmed (26). Together, these findings stress the importance of good hand hygiene and the use of surgical masks as mitigation strategies against respiratory droplets to prevent SARS-CoV-2 transmission (27). Reports also indicate that SARS-CoV-2 may follow alternative transmission routes (28, 29). Studies have shown a prolonged presence of SARS-CoV-2 viral RNA in fecal samples from infected patients. Urine and rectal swabs from children and adults have also been tested positive, even after negative nasopharyngeal tests, implying a risk of fecal-oral transmission (28, 29).

### Clinical Presentation

Typical clinical symptoms of COVID-19 disease include fever, dry cough, dyspnea, headache, and pneumonia. The clinical features revealed by chest computed tomography (CT) present as pneumonia, however abnormal features, e.g., alveolar damage, acute respiratory distress syndrome (ARDS), acute cardiac injury, and incidence of ground-glass opacities have also been reported (1, 30). The symptoms of COVID-19 infection appear after an incubation period of ~5.2 days (31). The period from the onset of symptoms until death ranges from 6 to 41 days with a median of 14 days (32), depending on the age, immune system status, and care of the patient, and it has been shown to be shorter for patients above 70 years of age (32). The CT findings and COVID-19 symptoms show similarities to infection with other betacoronaviruses, i.e., SARS-CoV and MERS-CoV. In addition, COVID-19 patients develop gastrointestinal symptoms like diarrhea, emphasizing the importance of testing fecal and urine samples to exclude any potential alternative transmission route (28, 33). A recent review by the Chinese Center for Disease and Prevention including 72,314 cases of COVID-19 showed that <1% of the cases represented children younger than 10 years of age (34).

## Immunopathology and Host Immune Response

### Innate Immune Response

**Figure 2:**
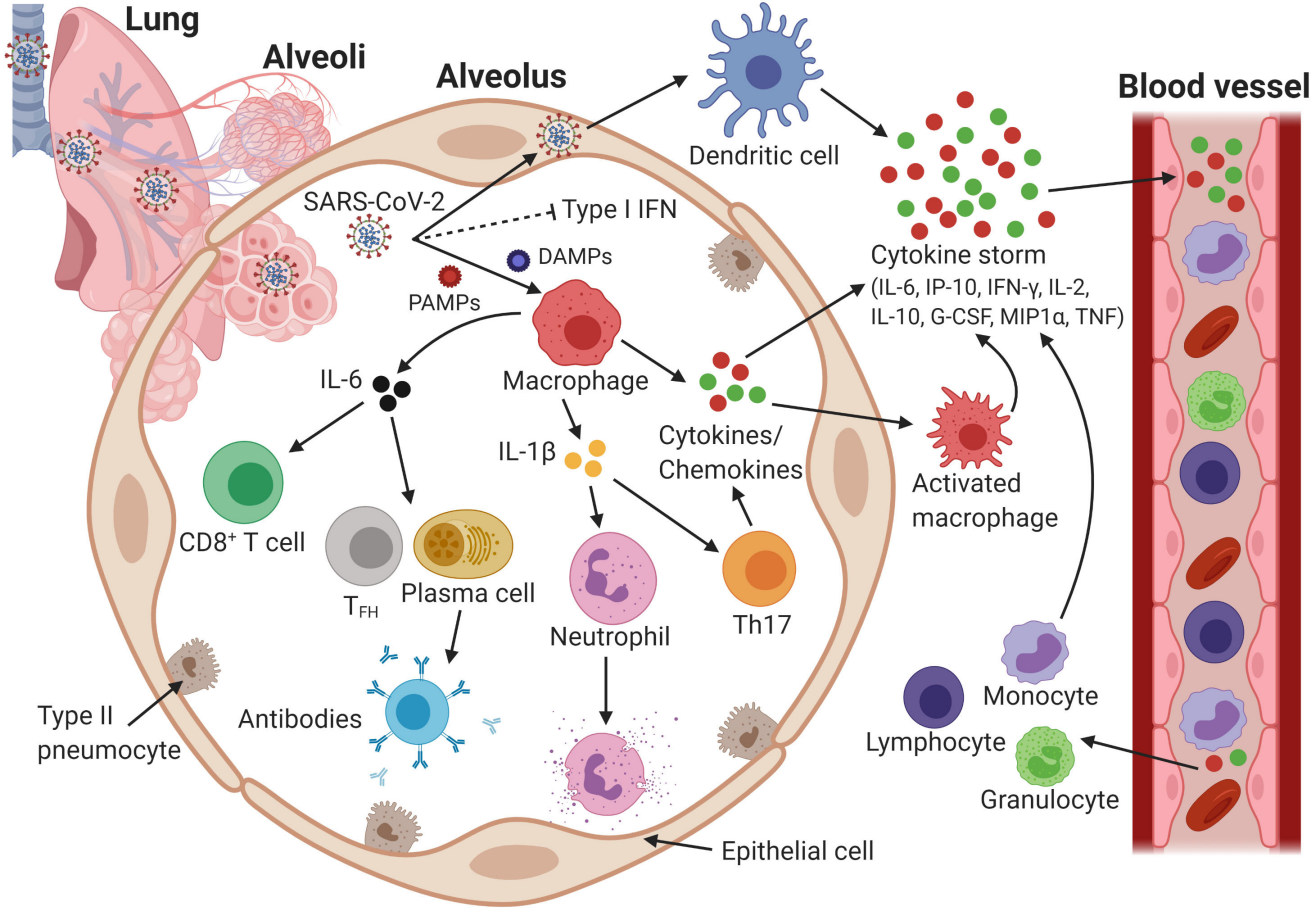
Host immune response and immunopathology during severe acute respiratory syndrome coronavirus 2 (SARS-CoV-2) infection. SARS-CoV-2 infects cells expressing the surface receptors angiotensin- converting enzyme 2 (ACE2) and transmembrane serine protease 2 (TMPRSS2). SARS-CoV-2 dampens anti-viral type I IFN responses, which results in uncontrolled viral replication. Viral pathogen-associated molecular patterns (PAMPs) and danger-associated molecular patterns (DAMPs) activate epithelial cells, endothelial cells, and tissue-resident macrophages to release proinflammatory cytokines and chemokines, including interleukin 6 (IL-6), IFN gamma-induced protein 10 (IP-10), IFN gamma (IFN-γ), IL-2, IL-10, macrophage inflammatory protein 1α (MIP1α), MIP1β, monocyte chemoattractant protein 1 (MCP1), granulocyte colony-stimulating factor (G-CSF), and tumor necrosis factor alpha (TNF-α). Cytokine- and chemokine-activated macrophages and virus-infected dendritic cells mediate extensive production of additional cytokines and chemokines, which eventually initiates a so-called cytokine storm. Chemokines attract more inflammatory cells that migrate from the blood vessels into the lungs, and these cells intensify the cytokine storm by releasing additional proinflammatory chemokines and cytokines, hence establishing a proinflammatory feedback loop. The cytokines circulate to other organs via the blood, eventually causing multi-organ damage. The downstream production of the cytokines IL-6 and IL-1β recruits neutrophils and CD8^+^ T cells, which not only control viral growth but also induce tissue damage, leading to alveolar flooding and consolidation (acute respiratory distress syndrome). IL-6 may recruit T-helper type 17 cells (Th17), which exacerbate inflammatory responses following activation. IL-6 also recruits follicular helper T cells (T_FH_) and B cells/plasma cells, which produce SARS-CoV-2-specific antibodies that may help virus neutralization. Alternatively, B cells produce non-neutralizing antibodies that enhance SARS-CoV-2 infection through antibody-dependent enhancement, which further exacerbate organ damage. Created with Biorender.com.

Currently, only limited data is available characterizing the innate immune response of patients against SARS-CoV-2. In one study from Wuhan, China, increased total numbers of neutrophils (38%), reduced total numbers of lymphocytes (35%), increased serum IL-6 levels (52%), and increased c-reactive protein levels (84%) were observed for 99 patients (1). In addition, a meta-analysis of six clinical studies conducted in China showed that the neutrophil-to-lymphocyte ratio was significantly increased in patients with severe COVID-19, whereas the lymphocyte-to-C-reactive ratio protein was significantly decreased (35). In a separate study, the numbers of T cells and CD8^+^ T cells were significantly lower, while the number of NK cells was reduced considerably in patients with severe COVID-19, as compared to the numbers for individuals with mild disease (36). Furthermore, an exuberant increase of the plasma levels of interferon gamma-induced protein 10 (IP-10), monocyte chemoattractant protein 1 (MCP-1), macrophage inflammatory protein 1α (MIP-1α), and tumor necrosis factor alpha (TNF-α) was associated with disease deterioration and a fatal outcome [(37); Figure 2]. These clinical features suggest a remarkably higher pro-inflammatory condition in the disease progression and severity than previously reported for SARS-CoV and MERS-CoV infection, suggesting a potential cytokine storm-mediated disease severity [(38); Figure 2].

Like SARS-CoV, SARS-CoV-2 exploits the receptor ACE2 to gain entry into cells. ACE2 is widely expressed in cardiopulmonary tissues and in hematopoietic cells, including monocytes and macrophages (38). To mount an antiviral response, innate immune cells recognize virus invasion by pathogen-associated molecular patterns (PAMPs), which in the case of RNA viruses is either viral genomic ssRNA or double-stranded RNA. This genomic RNA is recognized either by endosomal RNA receptors, including Toll-like receptor (TLR)-3 and TLR7, or by the cytosolic retinoid-inducible gene (RIG)/melanoma differentiation-associated gene 5 (MDA5) receptor (39). Following recognition, a downstream signaling cascade is activated, which in turn activates a number of transcription factors, i.e., nuclear factor κB (NF-κB), activator protein 1 (AP-1), IFN response factor 3 (IRF3), and IRF7, which is accompanied by their translocation into the nucleus. These transcription factors induce the expression of type I IFN (IFN-α and IFN-β) and pro-inflammatory cytokines, e.g., TNF and IL-1, and chemokines, e.g., C–C motif chemokine ligand 2 and C-X-C motif chemokine ligand 8, which comprise the first line of anti-viral immune defense (39). The binding of IFN to the IFNα/β receptor activates the Janus kinase-signal transducer and activator of transcription 1 (JAK-STAT) pathway, which brings the receptor-associated kinases JAK1 and Tyk2 into close proximity, eventually resulting in phosphorylation of STAT1 and STAT2. STAT1/2 form complexes with IFN regulatory factor 9 (IRF9), which subsequently translocate into the nucleus to initiate transcription of IFN-stimulated genes (ISGs) (39). Induction of a type I IFN response may be sufficient to inhibit viral replication and dissemination in the early stage of viral infection (40). However, the production of type I IFN (IFN-α and IFN-β), which constitute key antiviral mediators, is inhibited in COVID-19 patients (41, 42). Reportedly, coronaviruses have evolved several immune evasion mechanisms to restrict the early induction of type I IFN (43, 44).

### Adaptive Immune Response

Neutralizing antibodies (nAbs) induced by virus infection play a crucial role in controlling viral infection. For SARS-CoV-2, nAbs limit the infection at a later phase and prevent re-infection upon a future encounter with the virus (45). Recently developed SARS-CoV- and MERS-CoV-specific nAbs target the S1-RBD, S1- N-terminal domain (NTD) and S2 region, respectively, and block protein-receptor interaction and interfere with viral entry into the host cell, hence inhibiting viral infection (46). However, no SARS-CoV-2-specific nAbs have been reported so far. SARS-CoV nAbs with potential cross-reactivity and/or cross-neutralizing activity against SARS-CoV-2 infection are currently being identified (45) because SARS-CoV-2 is closely related to SARS-CoV, and the S proteins of the two different viruses display high sequence identity (1). Encouragingly, recent studies show that nAbs from convalescent SARS patients can block SARS-CoV-2 from entering target cells *in vitro*, which suggests potential cross-protective epitopes between the two viruses (1, 47).

T cell-mediated immune responses in SARS-CoV have been well-elucidated (48). Both CD4^+^ and CD8^+^ T-cells provide broad and long-term protection against coronavirus infections. CD4^+^ T cells promote the proliferation of virus-specific antibodies by activating T-cell dependent B cells, whereas CD8^+^ T cells are cytotoxic and kill virus-infected cells. In COVID-19 patients, a significant T cytopoenia was observed in circulating CD4^+^ and CD8^+^ T cells (49). Furthermore, a progressive increase in the PD-1^+^CD8^+^ and Tim-3^+^CD8^+^ subpopulation, which corresponds to exhausted T cells, was observed in symptomatic patients (49). In another study, the function of NK and CD8^+^ T cells was exhausted with the increased expression of natural killer group 2 member A (NKG2A) in COVID-19 patients (36). Recently, the decreased T cell proportion in patients with severe COVID-19 was associated with a down-regulated gene expression related to Th17 cell activation and differentiation (50). In one study investigating samples from convalescent SARS-CoV infected patients, a higher magnitude of CD8^+^ T cells, as compared to CD4^+^ T cells, was observed. Both CD4^+^ and CD8^+^ T cells from patients with severe disease displayed a central memory phenotype, as compared to the cells from patients with mild disease. Strong T-cell responses correlated with high titers of nAbs, while a Th2 type cytokine response (IL-4, IL-5, and IL-10) was detected in patients with a fatal outcome (51). The strong evidence that a Th1 type immune response plays a significant role in clearing SARS-CoV and MERS-CoV infection applies presumably also for clearance of SARS-CoV-2 infection. A recent study reported SARS-CoV-2-specific CD4^+^ T cells in all and CD8^+^ T cell responses in most COVID-19 patients (52). Importantly, this study also identified SARS-CoV-2-reactive CD4^+^ T cells in ~40–60% of unexposed individuals, which suggests cross-reactive T cell recognition between circulating “common cold” coronaviruses and SARS-CoV-2.

## Animal Models

Validated and predictive animal models represent important tools in the translation of vaccine candidates from bench to bedside because they help improving the understanding of disease biology and the requirements for developing of safe and efficacious vaccines. Validation of animal models is based on the criteria that animal models represent humans in terms of (i) comparable disease biology and clinical symptoms, i.e., face validity, (ii) displaying clinical interventions, which exhibit similar biological effect, i.e., predictive validity, and (iii) analogous function of the therapeutic target, i.e., target validity (53). An ideal animal model is immunocompetent and reproduces the typical features of human disease as closely as possible upon receiving a bio-relevant dose of challenge virus via an appropriate inoculation route (54).

Models based on mice, which are easy to breed and handle, often represent the animal models of choice in biomedical research, and murine models would be relevant for COVID-19 vaccine research. However, wild-type mice are resistant to SARS-CoV-2 infection because murine ACE2 is significantly different from the human receptor (1). However, genetically modified heterozygous mice that express both the murine and the human ACE2 receptor have been developed and used for testing of novel vaccine candidates during the SARS-CoV outbreak (55). Compared to wild-type mice that display only mild symptoms, transgenic mice expressing the human ACE2 receptor develop clinical illness after SARS-CoV-2 infection, including weight loss and interstitial pneumonia, and viral antigens have been detected in their bronchial epithelial cells, alveolar macrophages, and alveolar epithelial cells (56). However, the expression of the human ACE2 receptor in transgenic mice is not physiological, and transgenic mice are currently not readily available for testing of SARS-CoV-2 vaccine candidates. ACE2 knockout mice have been used in ARDS and SARS research and may also be useful for studying ARDS associated with COVID-19 (57). Transmembrane serine protease 2 (TMPRSS2) knockout mice may also be useful for investigating COVID-19 pathogenesis because TMPRSS2 is involved in cellular SARS-CoV-2 entry (58). In addition, STAT1 knockout mice develop progressive lung disease, including diffuse interstitial pneumonia and spread to other systemic organs, hence they may be useful for studying disease pathogenesis (1, 58). Adaptation of SARS-CoV by serial passage in the lungs of BALB/c mice resulted in a virus (MA15) that was lethal for young mice following intranasal inoculation and was preceded by high viral titer in the lungs, viremia, and spreading of virus to other systemic organs (59). With the availability of mouse-adapted SARS-CoV-2 isolates, it is expected that inbred mice could be useful to study the disease and evaluate novel vaccine candidates and antiviral drugs (60). Young inbred mice, for example of the strains BALB/c, C57BL/6, and 129S6, support SARS-CoV replication, but without development of disease, and these strains may be useful for evaluating immune responses to COVID-19 infection and vaccines (61). On the other hand, old (12–14 months) BALB/c mice exhibit patchy interstitial pneumonia following SARS-CoV infection, hence they can be used for COVID-19 research, especially to model the age-related higher mortality in humans (62). Aged C57BL/6 and 129S6 mice can also be used for these studies, but they exhibit lower viremia, as compared to BALB/c mice, following SARS infection (63). C57BL/6 mice have been used in SARS (64) and MERS (65) coronavirus-induced ARDS and can also be used for studying ARDS associated with COVID-19.

Ferrets have been widely used as a model for studying several respiratory viruses (66, 67). Viral replication has been detected both in the upper and lower respiratory tract of ferrets after infection with influenza and SARS-CoV (66–68). However, SARS-CoV-2 was found to replicate only in the nasal turbinate, soft palate, and tonsils of ferrets (69). SARS-CoV-2 can apparently also replicate in the digestive tract of ferrets, because viral RNA has been detected in the rectal swabs, but the virus was not detected in the lung lobes of ferrets, even after intratracheal inoculation (69). Between ferrets and humans, there is a difference of two amino acids in the segment of ACE2 to which SARS-CoV-2 first attaches (69), but the reason for the inability of SARS-CoV-2 to replicate in the lower respiratory tract of ferrets remains elusive. Despite this, the replication of SARS-CoV-2 in the ferret upper respiratory tract implies that ferrets represents an interesting animal model for evaluation of COVID-19 vaccine candidates.

Golden Syrian hamster is another widely used experimental animal model, which supports replication of SARS-CoV (63, 70) but not MERS-CoV, which uses the dipeptidyl peptidase−4 protein for viral entry (71). Golden Syrian hamsters represent a suitable experimental animal model for SARS-CoV−2 infection, because efficient viral replication takes place in the upper and lower respiratory epithelial cells, the animals display apparent clinical signs accompanied with weight loss, and high viral titers are found in the lungs and the intestine (72). Moreover, SARS-CoV-2 infection in Golden Syrian hamsters not only satisfies the Koch's postulates [(i) the pathogen must be present in every case of the disease, (ii) the pathogen must be isolated from the diseased host and grown in pure culture, (iii) the specific disease must be reproduced when a pure culture of the pathogen is inoculated into a healthy susceptible host, and (iv) the pathogen must be recoverable from the experimentally infected host] but also indicates virus transmission between challenged hamsters and naïve contact hamsters housed in the same cages (72). The differences in the susceptibility of mice and hamsters to SARS-CoV-2 infection are suggested to be related to the fact that in mice, 11 of the 29 amino acids present in the SARS-CoV-2 spike-contacting regions of ACE2 differ in the human ACE2 as compared to only four amino acids in hamster ACE2 (72). Nevertheless, in contrast to the large animal models and ACE2-transgenic mice, the Golden Syrian hamster model is easily available, physiologically relevant, and closely reflects COVID-19 infection, hence it represents a useful tool for studying the pathogenesis, treatment, and vaccines for COVID-19.

Despite being expensive, not readily available, and difficult to handle, non-human primates (NHPs) often represent the last stage of animal testing before any drug or vaccine candidate can enter clinical trials. NHPs are the gatekeepers for clinical trials due to their close genetic relationship with humans. Among the NHPs, African greens, rhesus macaques, cynomolgus macaques, and marmosets are being studied for SARS-CoV-2 infection. In one study including eight cynomolgus macaques, four of the oldest macaques excreted virus from the nose and the throat without any clinical signs after SARS-CoV-2 infection (73). The virus was detected in type I and II pneumocytes and in ciliated epithelial cells of the nasal, bronchial, and bronchiolar mucosa (73). In another study, two rhesus macaques that recovered from SARS-CoV-2 infection were reinfected after confirmed recovery, but they did not shown any signs of COVID-19 4 weeks later (74). This finding suggests a possible protection following natural infection or vaccination against COVID-19. In another study, older rhesus macaques infected with SARS-CoV-2 exhibited more severe interstitial pneumonia than younger macaques (75). This age-related difference in the pathogenicity of SARS-CoV-2 in NHPs may be useful for evaluation of therapeutics and vaccines due to the close correlation to humans. In a recent study, rhesus macaques were rechallenged with SARS-CoV-2 and displayed a 5 log_10_ reduction in the viral titers in the bronchoalveolar lavage and nasal mucosa, as compared to the primary infection, which suggests that the SARS-CoV-2 infection induces protective immunity against a subsequent exposure (76). Efforts are also underway to develop NHP models that can mimic the co-morbidities in COVID-19, e.g., hypertension and diabetes.

## Vaccine Platform Technologies

**Figure 3:**
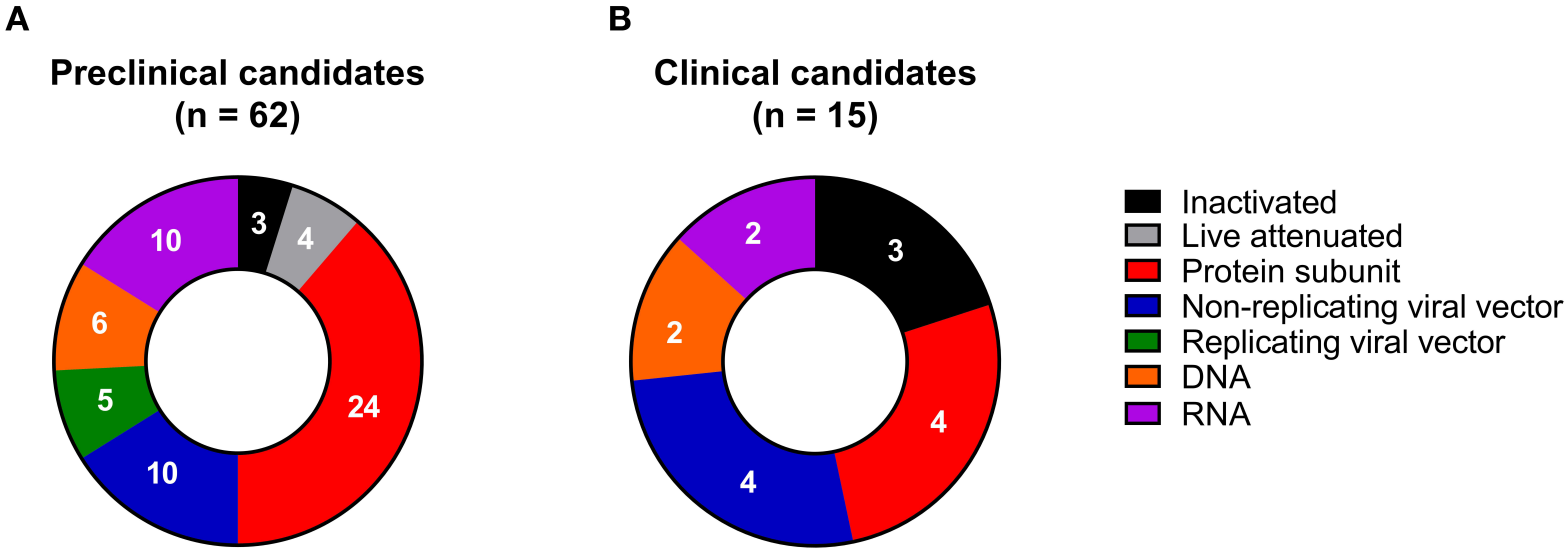
Examples of COVID-19 vaccine candidates in **(A)** preclinical (*n* = 62) and **(B)** clinical development (*n* = 15), grouped according to vaccine platform technology.

In the past decades, a wide array of novel vaccine platform technologies has been developed, thanks to advances primarily in molecular biology and vaccinology. These platform technologies range from inactivation and targeted attenuation of live pathogens to the delivery of synthetic peptide antigens and recombinantly produced protein antigens, as well as virus-like particles (VLPs), non-replicating and replicating viral vectors, polysaccharide-protein conjugates, and nucleic acid-based (DNA and RNA) vaccines. The existing marketed vaccines against infectious diseases are based on many of these platform technologies (77, 78). However, it is striking that all types of vaccine platform technologies are currently evaluated against COVID-19 in preclinical animal models (Figure 3A and Table 1), and some of them have even progressed into clinical development (Figure 3B and Table 2). This broad diversity increases the chances that at least a few of the candidates eventually will become approved and marketed.

**Table 1:** Examples of COVID-19 vaccine candidates in preclinical development.

**Vaccine platform**	**Vaccine candidate/Information**	**Developer**	**Status**	**Trial/Production site**	**Link**	**Reference to the technology**
**INACTIVATED**						
	Formalin-inactivated	Osaka University/BIKEN/National Institutes of Biomedical Innovation, Health and Nutrition (NIBIOHN)	Animal testing planned	Osaka, Japan	WHO	(79, 80)
	SolaVAX: Chemically inactivated	Colorado State University	Animal testing ongoing	Fort Collins, CO, USA	Colorado State University	(81–83)
	Inactivated vaccine + CpG 1018 adjuvant	Sinovac/Dynavax	Animal testing planned	Emeryville, CA, USA; Beijing, China	Dynavax	(84–86)
**LIVE ATTENUATED**						
	Viral de-optimized live attenuated vaccine	Codagenix/Serum Institute of India	Animal test results from mice and primates in August 2020	Farmingdale, NY, USA	Codagenix	(87, 88)
	Attenuated measles virus	German Center for Infection Research (DZIF)	Animal testing in mice in Autumn 2020	Brunswick, Germany	DZIF	(89, 90)
	Attenuated measles virus	Etna Biotech	Advancing preclinical candidate	Catania, Italy	Zydus Cadila	(91)
	Codon de-optimization technology	Griffith University/Indian Immunologicals	Ongoing animal testing	Brisbane, Australia; Hyderabad, India	Indian Immunologicals	–
**PROTEIN SUBUNIT**						
	Recombinant vaccine of SARS-CoV-2 S protein expressed in baculovirus system + pandemic adjuvant system (squalene, dl-α-tocopherol and polysorbate 80)	Sanofi Pasteur/GSK/Biomedical Advanced Research and Development Authority (BARDA)	Advancing preclinical candidate; clinical trial to begin between March and August 2021	Lyon, France; Brentford, UK; Washington, DC, USA	Sanofi Pasteur	(92–95)
	Molecular clamp-stabilized S protein	University of Queensland/GSK/CSIRO/ Viroclinics Xplore	Clinical testing in July, 2020	Queensland, Australia; Brentford, UK; Canberra, Australia; Rotterdam, The Netherlands	GSK University of Queensland	https://patentscope.wipo.int/search/en/detail.jsf?docId=WO2018176103; (96)
	COVID-19 XWG-03: truncated S protein	GSK/Xiamen Innovax Biotech Co., Ltd./Xiamen University	Advancing preclinical candidate	Brentford, UK; Xiamen, Fujian, China	GSK	(97–99)
	S protein	AJ Vaccines	Advancing preclinical candidate	Copenhagen, Denmark	AJVaccines	–
	S protein	Walter Reed Army Institute of Research (WRAIR)/U Army Medical Institute of Infectious Diseases	Ongoing animal testing	Maryland, United States	WRAIR	(100, 101)
	S protein	EpiVax/University of Georgia	Advancing preclinical candidate	Providence, RI, USA; Athens, GA, USA	EpiVax	(102–105)
	S protein	VIDO-InterVac, University of Saskatchewan	Ongoing animal testing	Saskatoon, SK, Canada	VIDO-InterVac	(106, 107)
	Adjuvanted S protein	National Institute of Infectious Disease	Advancing preclinical candidate	Tokyo, Japan	Japanese Agency for Medical Research and Development	(108, 109)
	PittCoVacc: Microneedle arrays S1 subunit	University of Pittsburgh	Clinical testing in Summer, 2020	Pittsburgh, PA, USA	University of Pittsburgh	(110)
	Recombinant protein, nanoparticles (based on S-protein and other epitopes)	Saint-Petersburg scientific research institute of vaccines and serums	Clinical testing in 2021	Saint-Petersburg, Russia	WHO	–
	Heat shock protein gp-96 backbone for multiple antigens	Heat Biologics/University of Miami	Advancing preclinical candidate	Morrisville, NC, USA; Miami, FL, USA	Heat Biologics	(111, 112)
	Receptor-binding domain (RBD) protein	Baylor College of Medicine/Texas Children's Hospital	Advancing preclinical candidate	Houston, TX, USA	Baylor College of Medicine	(113, 114)
	Adjuvanted RBD protein	Biological E Ltd.	Advancing preclinical candidate	Hyderabad, India	WHO	–
	DPX-COVID-19: Oil-based formulation with peptides epitopes of S protein	IMV Inc.	Clinical testing in Summer 2020	Québec, Canada	IMV	(115, 116)
	Human signal peptide domain complexed with undisclosed SARS-CoV-2 protein(s) as vaccine	Vaxil Bio Therapeutics	Advancing preclinical candidate (identified by *in silico* analysis)	Ness Ziona, Israel	Vaxi Bio Therapeutics	(117, 118)
	FlowVax COVID-19: Peptide, dry powder for injection or nasal spray	Flow Pharma Inc.	NHP testing in April 2020	East Palo Alto, CA, USA	Flow Pharma	(119, 120)
	Ii-Key hybrid peptide	Generex/EpiVax	Clinical testing in June, 2020	Toronto, Canada; Providence, RI, USA	EpiVax	(102, 103, 121, 122)
	Adjuvanted microsphere peptide	University of Saskatchewan	Ongoing animal testing	Saskatoon, SK, Canada	University of Saskatchewan	(123, 124)
	Synthetic long peptide vaccine candidate for S and M proteins	OncoGen	Advancing preclinical candidate	Timisoara, Romania	OncoGen	https://www.preprints.org/manuscript/202002.0102/v1
	Recombinant *Lactobacillus acidophilus* expressing S protein	Colorado State University	Advancing preclinical candidate	Fort Collins, CO, USA	Colorado State University	(125, 126)
	Drosophila S2 insect cell expression system virus-like particles (VLPs) (Split-protein conjugation system)	ExpreS^2^ion/Adaptvac/ University of Copenhagen	Clinical testing in April, 2021	Hørsholm, Denmark; Netherlands	ExpreS2ion/Adaptvac	(127–130)
	IBIO-200: Subunit protein (Virus-Like Particle), plant produced	iBio/CC-Pharming	Ongoing animal testing	Bryan, TX, USA; Beijing, China	iBio	(131, 132)
	VLP-recombinant protein administered with an adjuvant	Osaka University/BIKEN/NIBIOHN	Advancing preclinical candidate	Osaka, Japan	WHO	–
**NON-REPLICATING VIRAL VECTOR**						
	Ad26 (alone or with MVA boost)	Janssen Pharmaceutical Companies (Johnson & Johnson)/BARDA	Clinical testing in September 2020	New Jersey, USA	Johnson & Johnson	(76, 133)
	Modified Vaccinia Ankara encoded virus-like particles (MVA-VLP)	GeoVax/BravoVax	Ongoing animal testing	Atlanta, GA, United States; Wuhan, China	GeoVax	(134, 135)
	MVA-S encoded	DZIF—German Center for Infection Research	Animal testing in mice in Summer 2020	Brunswick, Germany	DZIF	(136, 137)
	AdCOVID: Adenovirus-based NasoVAX expressing SARS2-CoV S protein; nasal spray	Altimmune	Clinical testing in quarter three of 2020	Maryland, USA	Altimmune	https://www.ncbi.nlm.nih.gov/pmc/articles/PMC6253025/pdf/ofy209.162.pdf
	Ad5 S (GREVAX™ platform)	Greffex	Animal testing ongoing	Houston, USA	Greffex	
	SARS-CoV-2 protein VLP produced in tobacco	Medicago Inc.	Clinical testing in Summer 2020	Quebec, Canada	Medicago	(138, 139)
	Oral recombinant vaccine through adenovirus type 5 vector (Ad5)	Vaxart Inc.	Preclinical; Phase I in second half of 2020	San Francisco, USA	Vaxart	(140, 141)
	Adenovirus VLPs expressing SARS2-CoV S protein	Imophoron/University of Bristol	Animal testing planned	Bristol, UK	Imophoron	(142)
	Adenovirus vector expressing SARS2-CoV S protein	ReiThera/LEUKOCARE/ Univercells	Clinical testing in Summer 2020	Rome, Italy; Munich, Germany; Brussels, Belgium	ReiThera	(143, 144)
	Parainfluenza virus 5 expressing S protein	University of Georgia/University of Iowa	Animal testing ongoing	Athens, GA, USA; Iowa City, IA, USA	University of Georgia	(145)
**REPLICATING VIRAL VECTOR**						
	Measles vector	Institute Pasteur/Themis/University of Pittsburg Center for Vaccine Research	Animal testing planned	Paris, France; Vienna, Austria; Pittsburgh, PA, USA	Themis	(146, 147)
	TNX-1800: Horsepox vector expressing S protein	Tonix Pharma/Southern Research	Animal testing planned	Birmingham, AL, USA; New York, USA	Tonix Pharma	(148, 149)
	Vesicular stomatitis virus (VSV) vector expressing S protein	International AIDS Vaccine Initiative (IAVI)/Batavia Biosciences	Animal testing ongoing	New York, USA; Leiden, The Netherlands	IAVI	(150, 151)
	Influenza vector expressing RBD	University of Hong Kong	Clinical testing in July 2020	Hong Kong	University of Hong Kong	(152, 153)
	CoroFlu: Influenza virus expressing S protein	University of Wisconsin Madison/ FluGen/Bharat Biotech	Clinical testing in Fall 2020	Madison, WI, United States; Hyderabad, India	University of Wisconsin Madison	(154, 155)
**DNA**						
	DNA plasmid vaccine (electroporation)	Zydus Cadila	Advancing preclinical candidate	Ahmedabad, India	Zydus Cadila	–
	Four linear DNA-based vaccine candidates	Takis/Applied DNA Sciences/Evvivax	Preclinical testing in Autumn 2020	Stony Brook, USA; Rome, Italy	Evvivax	(156, 157)
	DNA	Osaka University/AnGes/Takara Bio	Animal testing in April 2020	Tokyo, Japan	AnGes	(158, 159)
	DNA with electroporation	Karolinska Institute/Cobra Biologics	Advancing preclinical candidate	Staffordshire, UK; Stockholm, Sweden	Cobra Biologics	(160, 161)
	Plasmid DNA, needle-free delivery	Immunomic Therapeutics, Inc./EpiVax, Inc./PharmaJet, Inc.	Animal testing ongoing	Rockville, MD, USA; Providence, RI, USA; Golden, CL, USA	Immunomix	(102, 103, 162, 163)
	DNA, nasal delivery	University of Waterloo	Advancing preclinical candidate	Waterloo, ON, Canada	University of Waterloo	(164, 165)
**RNA**						
	RNAoptimizer® technology	CureVac	Clinical testing in June 2020	Tubingen, Germany	CureVac	–
	mRNA	BIOCAD	Animal testing in April 2020	St. Petersburg, Russia	BIOCAD	–
	Lipid nanoparticle (LNP)-encapsulated mRNA	China CDC/Tongji University/Stermirna Therapeutics	Clinical testing in April 2020	Beijing, China	Xinhuanet.com	–
	LNP-encapsulated mRNA cocktail encoding VLP and LNP-encapsulated mRNA encoding RBD	Fudan University, Shanghai JiaoTong University, and RNACure Biopharma	Animal testing ongoing	Shanghai, China	Fudan University	http://chinaxiv.org/abs/202002.00070
	LNP-encapsulated saRNA	Imperial College London	Clinical testing in June 2020	UK	Imperial College London	(166, 167)
	LNP-encapsulated saRNA	Arcturus Therapeutics/Duke-National University of Singapore	Animal testing ongoing	San Diego, USA; Singapore	Arcturus Therapeutics	(168)
	mRNA for intranasal delivery	eTheRNA Immunotherapies/EpiVax/ Nexelis, REPROCELL/Centre for the Evaluation of Vaccination	Clinical testing in early 2021	Niel, Belgium	eTheRNA	(169, 170)
	mRNA	Sanofi Pasteur/Translate Bio	Animal testing planned	Lyon, France; Lexington, MA, United States	Sanofi Pasteur	(171, 172)
	Replication defective SARS-CoV-2 derived RNAs	Centro Nacional Biotecnología (CNB-CSIC)	Advancing preclinical candidate	Madrid, Spain	CNB-CSIC	(173, 174)
	LNP-encapsulated mRNA	University of Tokyo/Daiichi-Sankyo	Advancing preclinical candidate	Tokyo, Japan	Daiichi-Sankyo	(175)

**Table 2:** COVID-19 vaccine candidates in clinical trials.

**Study title**	**Vaccine**	**Sponsor**	**Location**	**Status**	**Phase**	**Primary outcome**	**Study identifier**
Safety and Immunogenicity Study of 2019-nCoV Vaccine (mRNA-1273) to Prevent SARS-CoV-2 Infection; Dose-Confirmation Study to Evaluate the Safety, Reactogenicity, and Immunogenicity of mRNA-1273 COVID-19 Vaccine in Adults Aged 18 Years and Older	mRNA-1273	National Institute of Allergy and Infectious Diseases (NIAID)/Moderna Therapeutics	Washington, USA	Recruiting	I; II	Relevant safety outcomes (12 months follow up); Adverse events (28 days post-vaccination); SARS-CoV-2-specific binding antibody (through 1 year after the final dose)	NCT04283461; NCT04405076
Immunity and Safety of Covid-19 Synthetic Minigene Vaccine	LV-SMENP-DC vaccine and antigen-specific CTLs	Shenzhen Geno-Immune Medical Institute	Guangdong, China	Recruiting	I/II	Clinical improvement based on a 7-point scale (28 days after randomization); Lower Murray lung injury score (7 days after randomization)	NCT04276896
Safety and Immunity of Covid-19 aAPC Vaccine	Pathogen-specific aAPC	Shenzhen Geno-Immune Medical Institute	Guangdong, China	Recruiting	I	Frequency of vaccine events; Frequency of serious vaccine events; Proportion of subjects with positive T cell response	NCT04299724
A Phase I Clinical Trial in 18-60 Adults (APICTH); A Phase II Clinical Trial to Evaluate the Recombinant Vaccine for COVID-19 (Adenovirus Vector) (CTII-nCoV); Phase I/II Clinical Trial of Recombinant Novel Coronavirus Vaccine (Adenovirus Type 5 Vector) in Canada	Recombinant Novel Coronavirus Vaccine (Adenovirus Type 5 Vector)	CanSino Biologics Inc./Institute of Biotechnology, China	Hubei, China; Halifax, Canada	Recruiting/Active, not recruiting; Not yet recruiting	I/II	Adverse reactions 0–7 days post-vaccination. Adverse reactions (0–14 days post-vaccination); IgG and neutralizing antibodies (28 days post-vaccination); Adverse reactions (0–6 and 0–28 days and 6 months after post-vaccination)	NCT04313127/ ChiCTR2000030906; NCT04341389; NCT04398147
A Study of a Candidate COVID-19 Vaccine (COV001) and Investigating a Vaccine Against COVID-19	ChAdOx1 nCoV-19	University of Oxford/Advent Srl	UK	Not yet recruiting	I/II and II/III	Efficacy, safety, and immunogenicity (6 months); Efficacy and safety (6 months)	NCT04324606 and NCT04400838
Evaluating the Safety, Tolerability and Immunogenicity of bacTRL-Spike Vaccine for Prevention of COVID-19	bacTRL-Spike (orally)	Symvivo Corporation	Canada	Not yet recruiting	I	Frequency of adverse events (up to 12 months post-vaccination)	NCT04334980
Safety, Tolerability and Immunogenicity of INO-4800 for COVID-19 in Healthy Volunteers	INO-4800 administered intradermally	Inovio Pharmaceuticals	Missouri and Pennsylvania, USA	Recruiting	I	Adverse events, injection site reactions, antigen-specific binding antibody titers and, IFN-γ responses (baseline up to week 28)	NCT04336410
Safety and Immunogenicity Study of 2019-nCoV Vaccine (Inactivated) for Prophylaxis SARS CoV-2 Infection (COVID-19); Safety and Immunogenicity Study of Inactivated Vaccine for Prevention of SARS-CoV-2 Infection (COVID-19)	Inactivated SARS-CoV-2	Sinovac Biotech Co., Ltd.	Jiangsu, China; Hebei, China	Recruiting; Not yet recruiting	I/II	Safety indexes of adverse reactions; Immunogenicity indexes of neutralizing-antibody seroconversion rates (up to 28 days after the whole schedule vaccination) Seroconversion rates of neutralizing antibody (30th day after the 2nd dose)	NCT04352608; NCT04383574
Study to Describe the Safety, Tolerability, Immunogenicity, and Potential Efficacy of RNA Vaccine Candidates Against COVID-19 in Healthy Adults; A Trial Investigating the Safety and Effects of Four BNT162 Vaccines Against COVID-2019 in Healthy Adults	BNT162 (BNT162a1, BNT162b1, BNT162b2) (Prime/Boost), BNT162c2 (Single Dose)	BioNTech RNA Pharmaceuticals GmbH and Pfizer	Mainz, Germany; Berlin, Germany	Recruiting; Recruiting	I/II	Solicited local reactions at the injection; Solicited systemic reactions (up to 7 ± 1 day after each immunization); Treatment-emergent adverse event (up to 21 ± 2 day after prime immunization and 28 ± 4 days after boost immunization)	NCT04368728; NCT04380701
Evaluation of the Safety and Immunogenicity of a SARS-CoV-2 rS (COVID-19) Nanoparticle Vaccine With/Without Matrix-M Adjuvant	SARS-CoV-2 rS and Matrix-M Adjuvant	Novavax	Victoria and Queensland, Australia	Not yet recruiting	I	Solicited adverse events (28 days); Serum IgG antibody levels specific for the SARS-CoV-2 rS protein antigen(s) (35 days)	NCT04368988
SCB-2019 as COVID-19 Vaccine	SCB-2019 with or without AS03 or CpG 1018 + Alum	Clover Biopharmaceuticals AUS Pty Ltd.	Australia	Not yet recruiting	I	Solicited adverse events (7 days after the first or second vaccination); Antibody Titers (Day 1 to Day 184)	NCT04405908
A clinical study for effectiveness and safety evaluation for recombinant chimeric COVID-19 epitope DC vaccine in the treatment of novel coronavirus pneumonia	Recombinant chimeric COVID-19 epitope DC vaccine	Shenzhen Third People's Hospital	Guangdong, China	Recruiting	I/II	Duration of disease; Antipyretic rate; Severe rate	ChiCTR2000030750
A randomized, double-blinded, placebo-controlled phase II clinical trial for Recombinant Novel Coronavirus (2019-nCOV) Vaccine (Adenovirus Vector)	Adenovirus type 5 vector vaccine	Jiangsu Provincial Center for Disease Control and Prevention	Jiangsu, China	Not yet recruiting	II	Adverse reactions 0–14 days post-vaccination; Anti-SARS-CoV-2 neutralizing antibody titer on day 28 post-vaccination	ChiCTR2000031781
A randomized, double-blind, placebo parallel-controlled phase I/II clinical trial for inactivated Novel Coronavirus Pneumonia vaccine (Vero cells)	Inactivated	Wuhan Institute of Biological Products Co., Ltd.	Wuhan, Hubei, China	Not yet recruiting	I/II	Incidence of adverse reactions/events (up to 7 days); Four-fold growth rate and antibody level, and cellular immunity (up to 90, 180, and 360 days)	ChiCTR2000031809
A phase I/II clinical trial for inactivated novel coronavirus (2019-CoV) vaccine (Vero cells)	Inactivated	Beijing Institute of Biological Products Co., Ltd.	Beijing, China	Recruiting	I/II	Incidence of adverse reactions/events (up to 7 days); Four-fold growth rate and antibody level (up to 28 days); Cellular immunity (Up to 28, 180, and 360 days)	ChiCTR2000032459

*aAPC, artificial antigen-presenting cell; COVID-19, coronavirus disease 2019; CTLs, cytotoxic T lymphocytes; DC, dendritic cell; LNP, lipid nanoparticle; mRNA, messenger RNA; nCoV, novel coronavirus; SARS-CoV-2, SARS coronavirus 2; S protein, SARS-CoV-2 spike protein. Sources: Chinese Clinical Trial Register website (www.chictr.org.cn); ClinicalTrials.gov website (www.clinicaltrials.gov); EU Clinical Trials Register (www.clinicaltrialsregister.eu)*.

### Inactivated Vaccines

Many approved vaccines are so-called inactivated vaccines based on inactivated pathogens, including the vaccines against polio, typhoid, cholera, plague, pertussis, and influenza. A few COVID-19 vaccine candidates based on this well-established technology are evaluated in preclinical studies [(79–81); Table 1]. This includes a formalin-inactivated COVID-19 vaccine candidate developed by Osaka University, Japan similar to their previous formalin-inactivated West Nile virus vaccine (79), which was found to be protective in mice and immunogenic in NHPs (80). Researchers at Colorado State University (Fort Collins, CO, USA) are developing an inactivated virus vaccine for COVID-19 (SolaVAX), which is based on an existing technology platform for pathogen inactivation in blood products including the use of ultraviolet light and riboflavin to inactivate the virus by targeted damage of nucleic acids while preserving the integrity of proteins and viral antigens (81, 82). This strategy has been shown to be efficient for inactivating MERS-CoV (83). Sinovac Biotech (Beijing, China) in collaboration with Dynavax (Emeryville, CA, USA) will evaluate the combination of Sinovac's chemically inactivated COVID-19 vaccine candidate (86) with Dynavax's advanced adjuvant CpG 1018 (84, 85). Sinovac is also testing their chemically-inactivated whole SARS-CoV-2 virus particles (PiCoVacc) developed in VERO monkey cells and the adjuvant alum (86) in phase I/II clinical trials (Table 2). PiCoVacc induced SARS-CoV-2-specific neutralizing antibodies in mice, rats, and NHPs and conferred complete protection in NHPs against SARS-CoV-2 (86). Using the same platform technology, candidate vaccines against influenza (176) and SARS (177) where shown to be safe and immunogenicity in phase I clinical trials. Wuhan Institute of Biological Products (Wuhan, China) and Beijing Institute of Biological Products (Beijing, China) are testing their vaccine candidates, which have been prepared by growing the SARS-CoV-2 in the VERO monkey cell line and inactivated with chemicals [(19); Table 2]. The development of conventional inactivated vaccines requires the cultivation of high titers of infectious virus, which in the case of SARS-CoV-2 has to take place in biosafety level 3 facilities, which is of major safety concern. Moreover, incomplete virus inactivation constitutes a potential risk to vaccine production workers and may also cause disease outbreaks in vaccinated populations and induce harmful immune or inflammatory responses.

### Live Attenuated Vaccines

Live attenuation of pathogens is yet another conventional vaccine technology, which is exploited for at least four novel COVID-19 vaccine candidates (Table 1). Live attenuated vaccines against several viruses have been commercialized, including influenza virus, rotavirus, polio virus, yellow fever virus, and measles virus. A live-attenuated vaccine has several advantages, including inducing an immune response against several different antigens of the virus and the possibility for scale-up for mass production. Codagenix (Farmingdale, NY, USA) and the Serum Institute of India (Pune, India) are co-developing a live-attenuated vaccine candidate against SARS-CoV-2 using rational, computer-aided gene design and chemical synthesis through a process referred to as *viral gene deoptimization* (87). A vaccine against respiratory syncytial virus designed using this technique has previously been shown to induce protective immunity in NHPs (88). The German Center for Infection Research (DZIF, Braunschweig, Germany) and Zydus Cadila (Etna Biotech, Ahmedabad, India) are developing a live attenuated recombinant measles virus (rMV) vectored vaccine against COVID-19. Using rMV, Etna Biotech has demonstrated the ability of a live attenuated human papillomavirus virus (HPV) vaccine to induce nAbs in NHPs (91), while DZIF has shown protection against infection with MERS-CoV (89) and Zika virus (90) in mice using this platform. Indian Immunologicals (Hyderabad, India) in collaboration with Griffith University (Brisbane, Australia) is exploiting the codon de-optimization technology to develop a live attenuated COVID-19 vaccine. Although live attenuated vaccines that target respiratory viral infections have been approved for use in humans, the fact that the virus is excreted in the feces of SARS-CoV-2-infected individuals (178, 179) generate concern that a live attenuated SARS-CoV-2 vaccine strain may also be excreted in the feces and can potentially transmit to unvaccinated individuals. Yet another potential matter of concern is the risk of recombination of a live attenuated vaccine virus with wild-type CoV.

### Subunit Vaccines

Subunit vaccines are based on synthetic peptide(s) or recombinant protein(s) of the target pathogen. Several approved vaccines are subunit vaccines, for example vaccines against HPV, hepatitis B virus and influenza virus. Unlike inactivated viruses, live attenuated viruses, and virus-vectored vaccines, subunit vaccines only contain specific viral antigenic fragments and do not include any additional components of the pathogenic viruses. Therefore, this approach eliminates the concerns of incomplete viral inactivation, virulence recovery, and pre-existing anti-vector immunity (180). Hence, subunit vaccines are generally considered very safe. In addition, subunit vaccines can specifically target well-characterized neutralizing antigenic epitopes and, in combination with adjuvants, improve immunogenicity, and/or efficacy (180). Because the S protein of SARS-CoV-2 plays a vital role in receptor binding and membrane fusion, vaccines targeting the S protein are suggested to be capable of inducing antibodies that can neutralize virus infection by blocking virus binding and fusion (181). Therefore, the S protein constitutes a major target antigen for SARS-CoV-2 subunit vaccine candidates (Table 1). However, in addition to the full-length S protein and its antigenic fragments, the S1 subunit, NTD, RBD, and the S2 subunit may also be important antigen targets for the development of subunit vaccines (20). Sanofi Pasteur (Lyon, France) and GlaxoSmithKline (GSK, London, UK) are developing a COVID-19 subunit vaccine candidate, where Sanofi contributes with an S-protein antigen, which is based on recombinant DNA technology using a baculovirus expression platform (92, 93). Using this platform, Sanofi has licensed a recombinant influenza vaccine in the USA (93). GSK contributes with a pandemic adjuvant technology based on the Adjuvant System 03 (AS03) comprising of squalene, dl-α-tocopherol, and polysorbate 80 (94, 95). GSK is also testing their adjuvant in collaboration with Clover Biopharmaceuticals, University of Queensland (Brisbane, Australia), and Xiamen Innovax Biotech (Xiamen, China). Utilizing its patented Trimer-Tag© technology, Clover Biopharmaceuticals has developed a SARS-CoV-2 S-Trimer subunit vaccine candidate SCB-2019 that resembles the native trimeric viral spike (182, 183). SCB-2019 is in phase I clinical testing with AS03 (94, 95) or CpG 1018 and alum adjuvants [(84, 85); Table 2]. Researchers at the University of Queensland are using the patented *molecular clamp* technology, which involves synthesizing a protein and subsequently clamping it onto virus-infected cells (184) as shown previously against flaviviruses (96). Molecular clamp-stabilized S protein will be combined with GSK adjuvants. COVID-19 XWG-03 is a preclinical vaccine candidate developed by Xiamen University (Xiamen, China) and Xiamen Innovax Biotech using the GSK adjuvant AS04 (monophosphoryl lipid A and aluminum hydroxide) (97). COVID-19 XWG-03 is based on a series of truncated S proteins, which will be screened in combination with AS04. Xiamen Innovax Biotech has previously developed similar *Escherichia coli*-produced subunit vaccines against HPV (98) and hepatitis E (99) in humans. The Walter Reed Army Institute of Research (Silver Spring, MD, USA) is also targeting the S protein and has previously demonstrated efficacy of a MERS-CoV S1-protein subunit vaccine in mice and NHPs (100), as well as in camels and alpacas (101). EpiVax (Providence, RI, USA) is exploiting the proprietary iVAX toolkit that comprises a suite of immunoinformatics algorithms for sorting candidate antigens, selecting immunogenic and conserved T cell epitopes, and eliminating regulatory T-cell epitopes (102–104). The optimized S-protein antigens will be tested for immunogenicity and protection against a SARS-CoV-2 challenge in collaboration with University of Georgia (Athens, GA, USA), which has previously tested the platform against influenza (105). Vaccine and Infectious Disease Organization—International Vaccine Centre (VIDO-InterVac, University of Saskatchewan, Saskatoon, Canada) is developing an S protein subunit vaccine based on prior experience with testing of a MERS-CoV vaccine candidate in NHPs (106, 107). The National Institute of Infectious Disease (Tokyo, Japan) is aiming at developing a new vaccine by combining an undisclosed adjuvant and an antigen using recombinant protein synthesis as previously demonstrated against influenza virus H5N1 (108, 109). PittCoVacc is a subunit vaccine candidate from University of Pittsburgh (Pittsburgh, PA, USA) that is based on a microneedle array (MNA) embedded SARS-CoV-2 S1 protein, which was recently found to elicit strong antigen-specific antibody responses for up to 2 weeks in mice (110). This MNA platform is currently tested in clinical trials against cutaneous T-cell lymphoma (*ClinicalTrials.gov Identifier: NCT02192021*). The COVID-19 vaccine candidate of Heat Biologics (Morrisville, NC, USA) is based on its secreted heat shock protein chaperone gp96 platform and has been shown to induce protection against simian immunodeficiency virus (SIV) in NHPs (111, 112). Vaccine researchers at Baylor College of Medicine and Texas Children's Hospital (both Houston, TX, USA) are using their experience with developing a SARS vaccine antigen consisting of the RBD of the SARS-CoV S protein to develop a similar vaccine against SARS-CoV-2 (113, 114).

In addition to vaccines based on full protein, several vaccine developers are investigating peptides antigens as vaccine candidates against COVID-19. IMV Inc. (Québec, Canada) is developing a vaccine candidate based on the IMV's DPX delivery technology and incorporating peptides targeting S protein epitopes of SARS-Cov-2 as shown previously for respiratory syncytial virus (RSV) (115) and anthrax (116). Vaxil Bio Therapeutics (Toronto, Ontario, Canada) is using the proprietary bioinformatic approach VaxHit™ to identify signal peptide domains of SARS-CoV-2 proteins as shown for mucin 1 tumor-associated antigen in mice (117) and in multiple myeloma patients (118). FlowVax COVID-19 is a candidate vaccine from Flow Pharma (Palo Alto, CA, USA) consisting of an adjuvanted, thermostable, and biodegradable peptide-loaded microsphere vaccine targeting the SARS-CoV-2 nucleocapsid. Flow Pharma has developed and tested a Zika virus vaccine candidate that induces cytotoxic T cell (CTL) responses in mice (119, 120). Generex Biotechnology (Toronto, Canada) is using EpiVax's computational tools to predict epitopes that can be used to generate peptide-based COVID-19 vaccines using the patented NuGenerex Immuno-Oncology Ii-Key technology (NGIO). NGIO technology has been used to develop peptide-based vaccine candidates, which have been tested against a HPV16+ cancer model in mice (121) and prostate cancer in humans (122). University of Saskatchewan's VIDO-InterVac is developing a peptide-based, microsphere-adjuvanted COVID-19 vaccine candidate using a combination adjuvant platform (TriAdj) (123) comprising of a TLR agonist (either polyinosinic-polycytidylic acid or CpG oligodeoxynucleotides), a host defense peptide, and polyphosphazene. TriAdj has been used to generate vaccine-induced protective immunity against several infectious diseases in animals and humans (124, 185). Colorado State University is developing a novel oral COVID-19 vaccine candidate using recombinant *Lactobacillus acidophilus* expressing the viral S protein. This platform has been shown to induce Th1 and Th17 responses against HIV-1 epitopes in mice after oral administration (125, 126). ExpreS^2^ion Biotechnology (Hørsholm, Denmark), Adaptvac (Hørsholm, Denmark), and University of Copenhagen is applying a *Drosophila melanogaster* Schneider 2 stable cell line expression system expressing VLPs to generate a novel vaccine candidate as previously used for malaria in human clinical trials (127, 128). They are utilizing this split-protein conjugation technology to generate stable isopeptide-bound antigen-VLP complexes by mixing antigen and VLP components. The technology has been demonstrated to induce broadly nAbs specific for HIV-1 V3 glycan in mice and macaques (129), and it has been used to develop a combinatorial HPV and placental malaria vaccine (130). iBio (Newark, DE, USA) in partnership with CC-Pharming (Beijing, China) is working on iBIO-200, which is a COVID-19 candidate vaccine based on Agrobacterium-mediated transient protein production in tobacco (Nicotiana benthamiana) plants and has been used for delivering recombinant proteins into mammalian cells (131) and for generating strong virus-specific nAb responses in animals (132).

Novavax (Gaithersburg, MD, USA) with support from the Coalition for Epidemic Preparedness Innovations (CEPI, Oslo, Norway), is clinically testing their COVID-19 subunit vaccine candidate prepared using the proprietary Sf9/baculovirus recombinant technology platform to generate S protein antigens as done previously for an RSV vaccine candidate (186). The protein antigens have been combined with a saponin-based Matrix-M™ adjuvant [(187); Table 2]. A subunit-based vaccine candidate from Shenzhen Third People's Hospital (Guangdong, China) in phase I/II testing is aimed at evaluating the effectiveness and safety of the recombinant chimeric COVID-19 epitope DC vaccine in the treatment of SARS-CoV-2-induced pneumonia.

### Non-replicating Viral Vector Vaccines

Viral vectors are used to deliver vaccine antigens to the target cells or tissues. A wide variety of replicating and non-replicating viral vectors are available. Adenoviruses and poxviruses represent examples of viral vectors, of which both replicating and non-replicating forms are available. Vectors designed primarily as replication-defective or non-replicating viral vectors include adeno-associated virus, alphavirus, and herpesvirus, while replicating vectors include measles virus, vesicular stomatitis virus, poliovirus, and yellow fever virus. Several of the non-replicating viral vector-based COVID-19 vaccine candidates in preclinical testing are based on adenovirus vectors (Table 1). Janssen (Johnson & Johnson, Leiden, The Netherlands) is using the AdVac® technology (based on adenovirus type 26) alone or in combination with the MVA-BN® technology based on a Modified Vaccinia Ankara (MVA) virus from Bavarian Nordic A/S (Hellerup, Denmark) as a prime-boost immunization approach against COVID-19. The adenovirus type 26 vector was demonstrated to mediate protection against SIV in NHPs (76) and immunogenicity against Ebola virus in clinical phase I testing (133). Vaxart (South San Francisco, CA, USA) initiated a project to develop a COVID-19 vaccine based on the VAAST^TM^ platform, which contains an adenovirus 5 vector and a TLR3 adjuvant, and it is designed as enteric-coated vaccine tablets that release the vector in the small intestine for targeted immune activation, as previously shown for an oral influenza candidate vaccine (140, 141). Imophoron's (Bristol, UK) in collaboration with University of Bristol (Bristol, UK) is using the ADDomer vaccine platform, which is an adenovirus-derived multimeric protein-based self-assembling nanoparticle scaffold engineered to facilitate plug-and-play display of multiple immunogenic epitopes from pathogens, and it has been tested against Chikungunya infection (142). ReiThera (Rome, Italy), LEUKOCARE (Munich, Germany), and Univercells (Brussels, Belgium) are developing a vaccine candidate based on ReiThera's simian adenoviral vector with strong immunological potency (143, 144) and Univercells's NevoLine™ biomanufacturing platform for scale up. GeoVax's (Atlanta, GE, USA) MVA platform technology has the advantage of being a live replication-competent vector in avian cells for manufacturing, yet replication-deficient in mammalian cells upon vaccination, and it was found to protect against Lassa fever virus in mice (134) and Ebola virus in NHPs (135). The DZIF is also developing a COVID-19 vaccine candidate based on MVA as a viral vector for the SARS-CoV-2 S protein, and protective efficacy against MERS infection has previously been demonstrated in mice (136) and camels (137). Medicago (Uppsala, Sweden) is using SARS-CoV-2 protein VLPs produced in tobacco (*Nicotiana Benthamiana*) to generate cellular and humoral immunity, as shown previously against influenza in clinical testing (138, 139). University of Georgia (Athens, GA, USA) in collaboration with University of Iowa (Iowa city, IA, USA) is developing a vaccine candidate using a parainfluenza virus 5 vector that encodes the S protein of SARS-CoV-2. Using this vector, a similar vaccine has been developed against MERS-CoV, which was protective in mice (145).

LV-SMENP-DC and pathogen-specific artificial antigen-presenting cell (aAPC) are the two lentiviral vector-based vaccine candidates in clinical trials from Shenzhen Geno-Immune Medical Institute (Guangdong, China) (Table 2). For the LV-SMENP-DC vaccine, an efficient lentiviral vector system (NHP/TYF) is used to express SARS-CoV-2 minigenes, engineered based on multiple viral genes, into viral proteins and immune-modulatory genes to modify DCs and to activate T cells (188, 189). In a similar strategy, a lentiviral vector system is used to express viral proteins and immune modulatory proteins to modify aAPC and to activate T cells (190). ChAdOx1 nCoV-19, developed by University of Oxford (Oxford, UK) and manufactured by Advent Srl (Pomezia, Italy), consists of an attenuated chimpanzee adenovirus capable of producing the S protein of SARS-CoV-2, and it is expected to induce antibodies against these proteins in SARS-CoV-2. The ChAdOx1 viral vector was shown to elicit nAbs and cellular immune responses in mice against human MERS-CoV (191). Another non-replicating viral vector-based vaccine candidate in clinical trials has been developed by CanSino Biologics (Hubei, China) and is based on a recombinant adenovirus type 5 vector (192).

### Replicating Viral Vector Vaccines

Measles virus, influenza virus, vesicular stomatitis virus, and horse pox virus, respectively, are used as replicating viral vector platforms to develop novel COVID-19 vaccine candidates (Table 1). Institut Pasteur (Paris, France) is exploiting their measles vaccine vector technology and has developed vaccine candidates against chikungunya (146) and MERS (147) based on this technology. Tonix Pharmaceuticals (New York, NY, USA) in collaboration with Southern Research (Birmingham, Alabama, USA) is developing TNX-1800, which is a live modified horsepox virus designed to express the S protein of SARS-CoV-2, and it is based on Tonix's biodefense vaccines against small pox and monkey pox (148, 149). The International AIDS Vaccine Initiative (IAVI, New York, NY, USA) is exploiting a recombinant vesicular stomatitis virus (rVSV) vector against COVID-19 and has demonstrated efficacy of rVSV-vectored vaccines against SIV in NHPs (150) and Ebola virus in humans (151). CEPI is partnering with The University of Hong Kong (Hong Kong, China) to develop a COVID-19 vaccine candidate based on a live-attenuated influenza vaccine platform (152, 153). The University of Wisconsin–Madison (Madison, WI, USA) and the vaccine companies FluGen (Madison, WI, USA) and Bharat Biotech (Hyderabad, India) have initiated the development and testing of the vaccine candidate CoroFlu that builds on the backbone of FluGen's flu vaccine candidate known as M2SR, which is a self-limiting version of the influenza virus in which gene sequences of SARS-CoV-2 are inserted to induce additional immunity against coronavirus (154, 155). Although several viral vector-based COVID-19 vaccine candidates are in preclinical as well as clinical development, several drawbacks are associated with the use of viral vectors to deliver genetic material to cells. First, the viral vector itself can induce an immune response in the body (193). Second, if a vaccine fails during clinical testing, the same viral vector cannot be reused in the patient because it can induce an immune response. Third, pre-existing immunity against the viral vector can render a vaccine ineffective (193). However, pre-existing immunity can be challenged by priming with a non-viral DNA vaccine (194) or by increasing the vaccine dose or changing the administration route (195). Other potential issues with viral vectors, e.g., low transgenic expression and genetic toxicity, can be overcome by using hybrid viral vectors (196).

### DNA Vaccines

This type of vaccine contains selected gene(s) of the virus in the form of DNA. Upon injection, the DNA is used as template for *in situ* expression of potentially harmless viral protein(s), which induces a protective immune response. One of the greatest advantage of this type of vaccine is the safety and scalability for mass production. DNA-based viral vaccines have been shown to induce strong immune responses in animal models, especially in mice (100, 197–199). However, there is limited positive clinical data on DNA-based viral vaccines in humans, and no commercial DNA vaccine against any disease has yet been approved. Nevertheless, several DNA vaccine candidates are tested preclinically (Figure 3A) and two candidates have progressed into phase I clinical testing (Figure 3B). Zydus Cadila (Ahmedabad, India) is developing a DNA vaccine against the S protein of SARS-CoV-2 based on an indigenously developed plasmid DNA delivery technology (19). Evvivax (Rome, Italy) collaborates with Applied DNA Sciences (Stony Brook, NY, USA) and Takis Biotech (Rome, Italy) to develop four linear DNA-based vaccine candidates. Evvivax utilizes viral or plasmid DNA vectors for *in vivo* delivery of an expression cassette carrying the coding region of the target gene in combination with the electro-gene-transfer technology from Takis Biotech (heterologous prime/boost) (156, 157). AnGes Inc. (Osaka, Japan) in partnership with Osaka University is developing a DNA-based COVID-19 vaccine candidate based on a hepatocyte growth factor plasmid, which has been used to develop a therapeutic DNA vaccine against hypertension (158, 159). Cobra Biologics (Newcastle, UK) and Karolinska Institutet (Stockholm, Sweden) are developing a DNA vaccine candidate, which is based on Cobra's ORT® (Operator-Repressor Titration) technology for producing plasmid DNA without antibiotics, antibiotic resistance genes or any other selectable marker genes (160, 161). These vaccine strategies all involve DNA administration by conventional intramuscular immunization. However, Immunomic Therapeutics (Rockville, MD, USA) is working with EpiVax and PharmaJet (Golden, CO, USA) to develop a DNA vaccine that is delivered intradermally using a needle-free injection system. This partnership will combine platform technologies from all three companies: Immunomic's (Rockville, MD, USA) UNiversal Intracellular Targeted Expression (UNITE) platform (162), EpiVax's *in silico* T-cell epitope prediction tool (102, 103), and PharmaJet's Tropis® needle-free injection system that accurately targets delivery to the intradermal tissue layer (163). Immunomic's UNITE platform involves fusing pathogenic antigens with lysosomal-associated membrane protein, which is an endogenous protein in humans, for enhanced MHC-II processing and MHC-I cross presentation and subsequent induction of both Th1 and CD8^+^ T cell responses (162). Researchers at the University of Waterloo (Waterloo, ON, Canada) are developing a DNA-based vaccine that is administered using a nasal spray. They will use a lambda bacteriophage system for delivering DNA into target cells (164, 165), which them produce SARS-CoV-2 VLPs that stimulate an immune response.

Among DNA vaccine candidates in clinical testing, bacTRL-Spike developed by Symvivo Corporation (Burnaby, Canada) is based on the bacTRL platform technology, which is a genetically modified live cell probiotic bacteria-based gene delivery platform (200). Each oral dose of bacTRL-Spike contains live *Bifidobacterium longum*, which has been genetically engineered to deliver plasmids containing synthetic DNA encoding the S protein of SARS-CoV-2. INO-4800 developed by Inovio Pharmaceuticals (Pennsylvania, USA) involves intradermal plasmid delivery directly into cells using INOVIO's proprietary hand-held device called CELLECTRA® 2000 (201, 202). The principle of CELLECTRA® 2000 is to use a brief electrical pulse to reversibly open small pores in the cell membrane to allow plasmid entry (203).

### RNA Vaccines

Similar to DNA vaccines, RNA vaccines contain selected genes of the virus in the form of mRNA, and following cytosolic delivery, these genes are translated into viral proteins. The mRNA-1273 from Moderna Therapeutics (Cambridge, MA, USA) is the first candidate vaccine that entered into Phase I clinical testing just 42 days after the sequencing of the full SARS-CoV-2 genome (*ClinicalTrials.gov identifier NCT04283461*) (Table 2). The mRNA-1273 has recently entered into phase II clinical testing (*ClinicalTrials.gov identifier NCT04405076*). mRNA-1273 is a novel LNP-encapsulated, mRNA-based vaccine that encodes the full-length, prefusion-stabilized S protein of SARS-CoV-2. This LNP-based technology platform has previously been shown to induce strong immune responses and protection against a number of different pathogens in preclinical studies (204, 205). In addition, the LNP technology was approved in 2018 for siRNA delivery as part of the product Patisiran (Onpattro, Alnylam Pharmaceuticals, Cambridge, MA, USA), which inhibits hepatocyte expression of transthyretin in patients with hereditary transthyretin-mediated amyloidosis (206). The mRNA-based vaccine candidate program BNT162 of BioNTech's (Mainz, Germany), which is developed jointly with Pfizer, is based on BioNTech's extensive experience with developing mRNA-based therapeutics, in particular against cancer, using customized mRNA molecules and intracellular delivery systems (207–209). BNT162 comprises of four vaccine candidates, each of which represent different mRNA formats and target antigens (S and RBD), and they are formulated using the LNP delivery system. Two candidates include nucleoside-modified mRNA (modRNA), one includes a uridine-containing mRNA (uRNA), and the fourth vaccine candidate is based on saRNA. CureVac (Tübingen, Germany) is exploiting the propriety RNAoptimizer® platform technology for developing a novel COVID-19 vaccine candidate (Table 1). BIOCAD (Saint-Petersburg, Russia) is designing an mRNA vaccine against SARS-COV-2 based on previous experience with mRNA-based cancer vaccines (19). The mRNA COVID-19 vaccine candidate co-developed by the Chinese Center for Disease Control and Prevention (Beijing, China), Tongji University School of Medicine (Shanghai, China), and Stermirna Therapeutics (Shanghai, China) is based on Stermirna's mRNA synthesis and lipopolyplex nano-delivery platform (19). Fudan University (Fudan, China), in cooperation with Shanghai JiaoTong University (Shanghai, China), and RNACure Biopharma (Shanghai, China), is pursuing two different strategies to develop mRNA vaccines against COVID-19. The first strategy includes an mRNA encoding the RBD of the S protein to induce nAbs (19), while the second strategy includes an mRNA that instructs the host to produce VLPs (19). Imperial College London (London, UK) is developing an mRNA COVID-19 vaccine based on prior work with lipid nanoparticle (LNP)-encapsulated self-amplifying RNA, which has previously been shown to induce antibodies against the HIV-1 Env gp140 (166, 167). Arcturus Therapeutics (San Diego, CA, USA) in collaboration with Duke National University of Singapore (Singapore) is developing a vaccine candidate using its STARR™ (self-transcribing and replicating RNA) technology platform that combines self-replicating RNA with the nanoparticle delivery system LUNAR® into a single solution for *in situ* expression of SARS-CoV-2 proteins that induce an anti-viral immune response (168). eTheRNA Immunotherapies (Niel, Belgium) is developing a novel vaccine using the proprietary TriMix technology platform (169, 170). The TriMix platform comprises three different mRNAs encoding proteins (caTLR4, CD40L, and CD70) that stimulates dendritic cells (DCs) to activate strong CD4^+^ and CD8 T^+^ cell responses, and it was shown to induce immunogenic responses in preclinical (169) and clinical studies (170) of an mRNA-based melanoma vaccine. Sanofi Pasteur and Translate Bio (Lexington, MA, USA) are collaborating to develop a novel mRNA vaccine based on Translate Bio's proprietary mRNA therapeutic platform (MRT^TM^). This platform includes the design of the desired mRNA sequences and then packaging them into delivery systems (171), and it has been shown to induce therapeutic antibodies against human epidermal growth factor receptor 2-positive tumors in humanized mice (172). Centro Nacional de Biotecnología (Madrid, Spain) is developing an mRNA COVID-19 vaccine candidate based on the highly attenuated poxvirus vector MVA expressing the S protein, which has previously been tested as a vector for vaccine candidates against Zika and Ebola viruses (173, 174). Daiichi Sankyo (Tokyo, Japan) is developing an mRNA vaccine encoding the S protein using their novel nucleic acid delivery technology based on LNPs, and the protective effects of the vaccine will be verified in animal models in partnership with University of Tokyo (Tokyo, Japan) (175).

### Repurposed Vaccines

Bacillus Calmette–Guérin (BCG), which is a live attenuated vaccine that was developed against tuberculosis, has been reported to decrease the susceptibility to respiratory tract infections (210, 211) through reprogramming of innate immunity (212). Currently, there is no evidence that the BCG vaccine affords protection against COVID-19. However, several phase III and IV clinical trials are investigating if the BCG vaccine can help to boost the immune system and reduce the infection rate of SARS-CoV-2 (*ClinicalTrials.gov identifier NCT04327206, NCT04348370, NCT04350931, NCT04362124, NCT04369794*, and *EU Clinical Trials Register 2020-001591-15, 020-001678-31*) or reduce absenteeism among healthcare workers involved in COVID-19 patient care (*NCT04328441* and *NCT04373291*). One phase I trial in China is testing the effect of inhalation of inactivated *Mycobacterium vaccae* on protection against COVID-19 (*Chinese Clinical Trials Register ChiCTR2000030016*).

## Vaccine Manufacture

Vaccine development and manufacture of sufficient doses to induce herd immunity is one of the most challenging tasks within biopharmaceutical enterprises due to the complexity of the products. The most basic requirements for manufacturing vaccines in a way that is safe, effective yet consistent from batch to batch are difficult to implement. A number of variables dictate the outcome of vaccine production processes, including (i) the biological variability of the starting material, (ii) the pathogen, (iii) the environmental conditions during culture, (iv) the expertise of the manufacturing personnel, and (v) multiple steps during the purification process. In addition to these variables, the analytical methods used and the antigens produced during manufacturing often have high intrinsic variability. Scale up and safety of vaccine formulations are equally important for maintaining a successful production process. Therefore, improved technologies to streamline vaccine development and manufacturing are crucial. During the past decades, multiple novel platforms have been developed for producing vaccines at pandemic speed, including VLPs, viral vector-based vaccines, and nucleic acid-based vaccines. Each platform has its own advantages and challenges related to its ability to induce potent immune responses, manufacturing capacity, and safety for clinical use (Table 3). Therefore, it is unlikely that any single platform on its own will constitute a solution for the ongoing COVID-19 pandemic or a pandemic situation in the future (213).

**Table 3:** Vaccine platform technologies used for developing vaccines against COVID-19.

**Platform**	**Antigen type**	**Immune response**	**Advantages**	**Disadvantages**	**Response time in pandemics**
Inactivated (egg-based)	Inactivated pathogen	Humoral Cellular	Over 70 years of experience Potent Simple formulation	Labor-intensive Difficult to manufacture in a short time Stringent quality control	Low
Live attenuated	Attenuated pathogen	Humoral Cellular	Potent Multivalent by nature Simple formulation No adjuvants required	Labor-intensive Difficult to manufacture in a short time Stringent quality control Risk for infection	Low
Subunit/Recombinant protein	Protein	Humoral	Non-infectious Less side effects	Labor-intensive New production process and stability assays for each new antigen Quality control Cold chain transfer and storage Need for adjuvants	Medium
Virus-like particles (VLPs)	Protein	Humoral	Non-infectious Potent	Stability Quality control Potential contaminants Assembly into stable particles Heterogeneity Cold chain transfer and storage	Medium
Viral vectors	Nucleic acid	Humoral Cellular	Potent No need for an adjuvant Antigens are expressed natively	Recombination of virus during production Contaminants from human- or animal-derived material Pre-existing immunity against the vector	High
DNA	Nucleic acid	Humoral Cellular	Room temperature storage Rapid large-scale production Options for multivalency Cell-free No contaminants Non-infectious	Weak immunogenicity in humans Risk of carcinogenesis due to potential genetic integration Difficult to scale up to g-kg scale Purity High concentration	High
mRNA	Nucleic acid	Humoral Cellular	Room temperature storage Ease of large-scale production Options for multivalency Cell free No contaminants Non-infectious No genome integration risk No anti-vector immunity	Scale up of mRNA synthesis Stability Stringent RNase-free environment Relatively higher cost Risk of adverse reaction Inflammation	High

## Concluding Remarks

Currently, SARS-CoV-2 is spreading and posing a considerable economic and public health concern globally. It is urgent to design and develop safe and efficacious vaccines to prevent further spread of COVID-19 and establish vaccine-induced herd immunity. The development of transforming vaccine technology platforms over the past few decades has broadened the scope and shortened the time from pathogen identification to the deployment of vaccine candidates for clinical testing. The global COVID-19 vaccine pipeline is currently expanding on a daily basis, and radical rethinking of vaccine development and manufacturing processes may substantially improve our responses to the COVID-19 pandemic. The knowledge generated through vaccine development efforts for closely related coronavirus strains, e.g., SARS and MERS, are used to direct the vaccine development efforts for COVID-19. Although inducing nAbs against the S proteins represents the main target for the majority of the vaccine candidates, the prospects of exploiting T and B cell responses for COVID-19 vaccination should also be considered, because these responses have been found to be persistent and protective in animal models. In addition, strategies worth further investigation include (i) vaccine potentiation with adjuvants, (ii) tailoring of S protein, (iii) targeting RBD and N proteins, (iv) mucosal immunization, and (v) the employment of unchartered vaccine platforms for reducing vaccine development time and costs, and/or for improving vaccine safety and efficacy. The lack of naturally acquired immunity against SARS-CoV-2 should not be considered a bottleneck in developing efficacious vaccines against COVID-19, because this was disproved for vaccination against now eradicated smallpox. Also, research efforts should be directed toward studying SARS-CoV-2 infection in appropriate animal models to analyze (i) viral replication, (ii) transmission, (iii) pathogenesis, and (iv) host immune responses, as well as (v) the effect of serious underlying medical conditions like hypertension and diabetes. To date, no mRNA vaccines have been licensed for human use. However, important lessons from the current advancement of the mRNA vaccine platform technology for COVID-19, which takes place at an unprecedented pace, may benefit the development of any target vaccine in the future, because the technology implies a significantly reduced overall time from target identification to regulatory approval and deployment of the vaccine. Collectively, the broad array of platform technologies under investigation in the development and manufacture of novel vaccines against SARS-CoV-2 will hopefully result in one or a few safe and efficacious novel COVID-19 vaccines that can bring us closer to the goal of COVID-19 herd immunity.

## Author Contributions

LF, YZ, and AT wrote the review article. CF and AT critically revised all versions of the article. All authors contributed to the article and approved the submitted version.

## Conflict of Interest

The authors declare that the research was conducted in the absence of any commercial or financial relationships that could be construed as a potential conflict of interest.
